# Method for fetal ultrasound image classification using pseudo-labelling with PCA-KMeans and an attention-augmented MobileNet-LSTM model

**DOI:** 10.1016/j.mex.2025.103563

**Published:** 2025-08-11

**Authors:** Aniket K. Shahade, Priyanka V. Deshmukh, Pritam H. Gohatre, Kanchan S. Tidke, Rohan Ingle

**Affiliations:** a Symbiosis Institute of Technology, Pune Campus, Symbiosis International (Deemed University), Pune, India; b Visvesvaraya National Institute of Technology, Nagpur, India; c Dr. Rajendra Gode Institute of Technology & Research, Amravati, India

**Keywords:** Fetal ultrasound, Image classification, Pseudo-labeling, PCA, K-means clustering, MobileNet, multi-head attention, LSTM, Deep learning, Transfer learning

## Abstract

Accurate classification of fetal ultrasound images is critical for early diagnosis, yet remains challenging due to limited labeled data and high inter-class variability. This study presents a robust deep learning framework that combines a MobileNet backbone with multi-head self-attention and LSTM layers to enhance feature learning and temporal context. To address data scarcity and imbalance, unsupervised clustering was employed using Principal Component Analysis (PCA) for dimensionality reduction and K-means (k=4) for pseudo-label generation. These pseudo-labeled clusters were then balanced using oversampling techniques. The proposed model was trained using transfer learning on the augmented dataset and achieved a test accuracy of approximately 98 % with a macro-F1 score of 0.98, indicating highly reliable classification performance.

• Employed PCA (100 components) and K-means (k=4) for effective pseudo-labeling and class balancing.
• Designed a hybrid deep learning architecture using MobileNet, multi-head attention, and LSTM.
• Achieved ∼98 % test accuracy and 0.98 macro-F1 score, demonstrating strong model generalization.

## Graphical abstract

**Figure:**
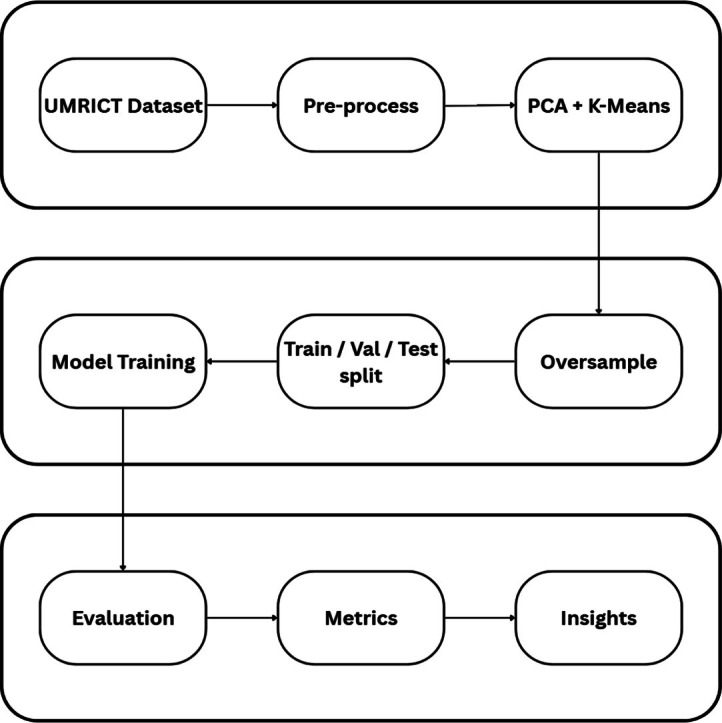

**Specifications table**

**Table:** 

**Subject area**	Computer Science
**More specific subject area**	Medical Image Analysis
**Name of your method**	Attention-MobileNet-LSTM with Pseudo-Labeling
**Name and reference of original method**	None
**Resource availability**	**UMRICT (Kaggle)**https://www.kaggle.com/datasets/shuvokumarbasak2030/medical-imaging-fetal-colorized-new-dataset-umrict

## Background

Fetal ultrasound imaging is of great importance in prenatal care because it helps to monitor the health of the fetus, identify possible problems, and predict complications during pregnancy. It is instrumental for timely diagnosis and adequate treatment that fetal ultrasound images are accurately and timely classified. Still, manual image review can be cumbersome, and is very much dependent on the screening abilities of the radiologists. Over the past few years, the way the deep learning approaches, including convolutional neural networks (CNNs) and recurrent neural networks (RNNs) have shown promise in automation of the medical image classification including the ultrasound based data [1].

For supervised learning to work, large volumes of labelled data are required, which is not very common since this premium clinical data requires many resources and effort. People have exploited pseudo-labelling, a reliable semi-supervised approach, to enable models to generate labels for unlabeled pictures during the training process, as proposed in previous research [2]. Research in image classification [3] shows that the use of pseudo-labeling in combination with clustering algorithms such as K-means enhances not only the accuracy of the label but also the capability of a model to deal with a wide range of data input.

The introduction of attention mechanisms to deep learning has greatly enhanced its use to yield a very good performance on sequential and spatial data. Using an effective architecture that is appropriate for mobile devices coupled with attention mechanisms and LSTM networks, MobileNet has been found to augment the model’s ability to determine important image features [4]. The optimized design of MobileNet makes it an excellent solution for medical image classification in environments constrained by resources [5].

Studies indicate that combining deep learning technique with targeted improvements hold a great promise in classifying ultrasound images. A possible solution is the use of transfer learning combined with pre-trained Convolutional Neural Networks (CNNs), such as MobileNet, to classify medical images with little labeled data [6]. It has been discovered that models can profit from attention mechanisms because they learn to concentrate on important parts of an image such as organs or abnormal regions, which improves their ability in classification tasks [7].

Further developments in ultrasound image classification are being made, but the problem of preserving accuracy on different datasets, addressing class imbalance and stopping overfitting are yet to be solved [8]. Early stopping and weight restoration methods are often used as effective methods of overfitting control during model training [9].

This study proposes an original approach to fetal ultrasound image classification combining pseudo-labeling, PCA-K Means, and an attention-augmented MobileNet-LSTM network. By combining these strategies, the proposed method seeks to improve the accuracy of classification, increase general applicability, and reduce overfitting for the task of classification of fetal ultrasound images.

The use of deep learning methods has significantly improved medical image classification accuracy over the last several years. By enabling models to draw attention to important image portions, attention mechanisms cause the interpretability and the accuracy to improve. Attention mechanisms applications in the contemporary frameworks of deep learning have shown impressive results in such medical areas as fetal ultrasound image interpretation [10]. And when medical images are interpreted by attention networks that focus on important anatomical structure and their spatial correlations, errors are less likely [11].

Integrating the lightweight efficient nature of MobileNet and CNNs and LSTM for processing sequential data is becoming a favorite solution in medical image classification workflows. The fast performance of MobileNet and the sequential processing of LSTM make such a framework particularly suitable for dynamic medical tasks involving images, such as fetal ultrasound [12]. The use of models of this type in combination with tools such as PCA allows preservation of crucial information while reducing computational demand and complexity [13].

Using deep learning models in combination with attention mechanisms has greatly improved the accuracy in ultrasound image classification, especially for extensive and diverse data sets. Adopting this technique helps to overcome the standard issues connected with image quality as noise, artifacts [14]. Computing power advances have also made lightweight models, such as MobileNet, and coupled with techniques such as early stopping, more popular for practical deployment of medical image classifiers in real world scenarios [15].

Recent advances in self-supervised learning (SSL) and contrastive learning have demonstrated strong performance in fetal ultrasound classification, offering alternative strategies to pseudo-labeling. For instance, [16] proposed a contrastive learning framework for standard-plane classification, leveraging unlabeled data to learn discriminative features without explicit pseudo-labels. Similarly, [17] introduced contrastive prototype federated learning to address label noise in fetal plane detection, achieving robustness comparable to supervised methods. While these approaches eliminate the need for clustering-based pseudo-labeling, they require large-scale pretraining and careful negative sampling. In contrast, our PCA-KMeans pseudo-labeling offers a lightweight, interpretable alternative suitable for smaller datasets, while still benefiting from semi-supervised principles. The proposed hybrid MobileNet-LSTM architecture further distinguishes our work by integrating temporal modeling, which is underexplored in SSL-based fetal ultrasound analysis.

## Method details

### Dataset

The color-enhanced ultrasound collection “Medical Imaging Fetal Colorized New Dataset – UMRICT” was downloaded from Kaggle (5 GB, 12662 PNG/JPG files, 224 × 224 px native resolution). Images have been organized into 13 augmentation-method folders. A Python script enumerating every file path and its respective folder name stored the result in two column pandas DataFrame (image_path, label) with 12,662 non-null rows and no duplicates.

The classes are named:

• 3D_Rendering
• 3D_Volume_Rendering
• Adaptive_Histogram_Equalization
• Alpha_Blending
• Basic_Color_Map
• Contrast_Stretching
• Edge_Detection
• Gamma_Correction
• Gaussian_Blur
• Heatmap_Visualization
• Interactive_Segmentation
• LUT_Color_Map
• Random_Color_Palette

Transparency reports were printed concerning any missing values, the respective data type, and memory footprint. Visualization in the count plot and pie chart ensured label distributions were intact, while the 5-image montages per category supported a qualitative inspection of color fidelity without any bias.

**Fig. 1:**
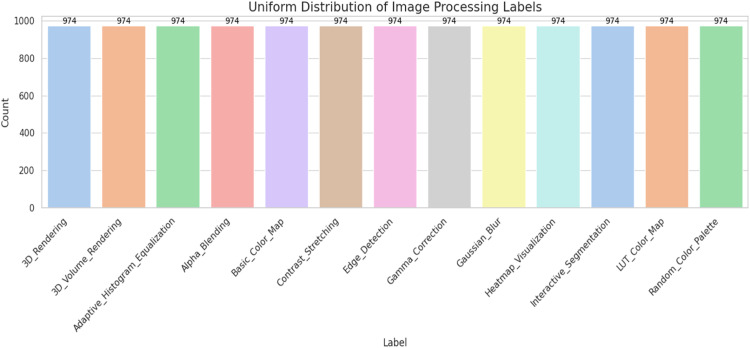
Uniform distribution of image processing labels in the dataset.

Fig. 1 illustrates the uniform distribution of image processing labels across 13 categories, with each class containing exactly 974 samples. This balanced dataset ensures that the classification model is trained on an equal number of instances per label, minimizing the risk of bias toward any specific class. Such uniformity is particularly beneficial for deep learning models, as it promotes stable convergence and enhances overall performance across all categories.

**Fig. 2:**
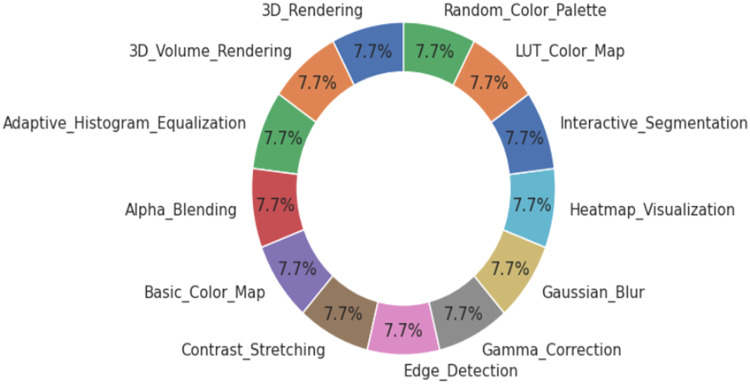
Proportional distribution of image processing labels.

Fig. 2 illustrates a pie chart representing the proportional distribution of image processing labels across the dataset. Each label, such as 3D Rendering, Gamma Correction, and Edge Detection, accounts for approximately 7.7 % of the total, confirming that the dataset is uniformly balanced across all 13 categories. This even distribution ensures unbiased training and evaluation of machine learning models, reducing the risk of overfitting to dominant classes. Such uniformity is particularly beneficial when benchmarking classification performance across diverse image processing tasks.

**Fig. 3:**
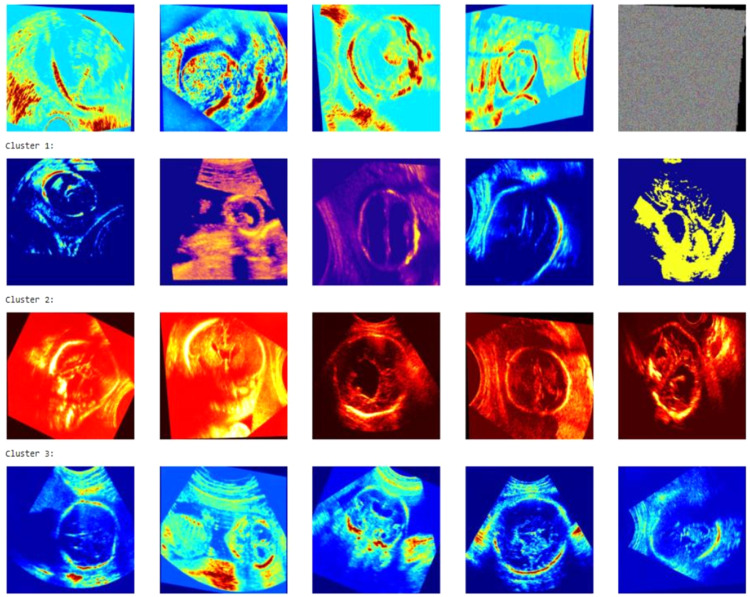
Visualization of image clusters based on feature similarity.

Fig. 3 presents sample visualizations from three distinct clusters of ultrasound images, generated using unsupervised clustering techniques such as K-Means. Each cluster groups images with similar texture, color mapping, and structural patterns, indicating underlying feature similarities captured by the model. Cluster 1 consists mostly of images with high contrast and edge-like patterns, Cluster 2 is dominated by intensely red-tinted heatmaps indicating aggressive transformations, while Cluster 3 shows images with more natural or moderate color distributions. This clustering approach is useful for understanding the latent structure of the dataset and can aid in anomaly detection, data curation, and pre-labeling tasks for downstream supervised learning.

### Unsupervised visual grouping

i. Vectorization and PCA:
  First, the image was resized to 64 × 64 RGB and flattened at a pixel intensity of [0, 1]. An image is also reduced to present the data in the dimension of 12 288-D. Principal-component analysis (PCA, n_components = 100) retained almost ≈ 95 % variance while dimensionality reduced by 120 folds.
ii. K-Mean Clustering: An elbow curve on distortion from k= 1 to 14 suggested k=4 as the inflection point.The KMeans (n_clusters=4, random_state=42) partitions the 100-D vectors into four latent clusters of different sizes given as:
  • 2626
  • 4379
  • 972
  • 4685
iii. Qualitative validation was performed by plotting five random thumbnails from each cluster.

**Fig. 4:**
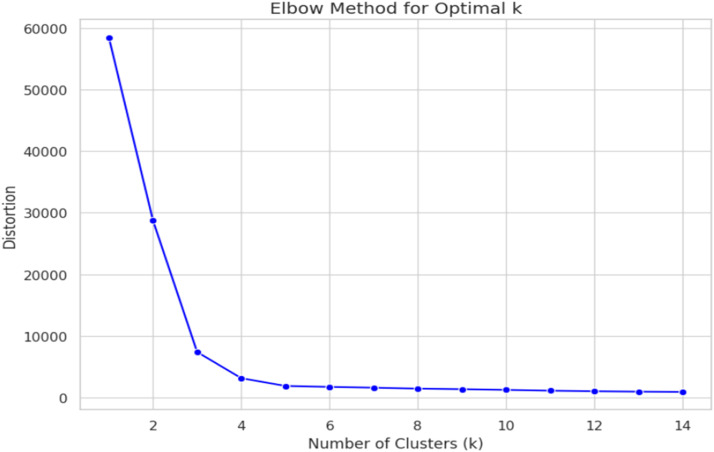
Elbow method to determine the optimal number of clusters (k).

Fig. 4 illustrates the Elbow Method, a popular technique used to identify the optimal number of clusters (k) in K-Means clustering. The X-axis represents the number of clusters, while the Y-axis shows the corresponding distortion or within-cluster sum of squares. As the number of clusters increases, distortion decreases. However, after a certain point (the "elbow"), the rate of improvement sharply diminishes. In this case, the elbow appears around k=3, suggesting that three clusters would balance compactness and simplicity in the data structure.

The elbow method shown in Fig. 4 indicated k=4 as the optimal cluster count, balancing distortion reduction and model simplicity. While this doesn’t align with anatomical categories, the clusters captured latent technical similarities (e.g., high-contrast vs. color-mapped images) that transcended anatomical boundaries. This approach proved effective for semi-supervised learning, as evidenced by the 98 % F1 score.

**Fig. 5:**
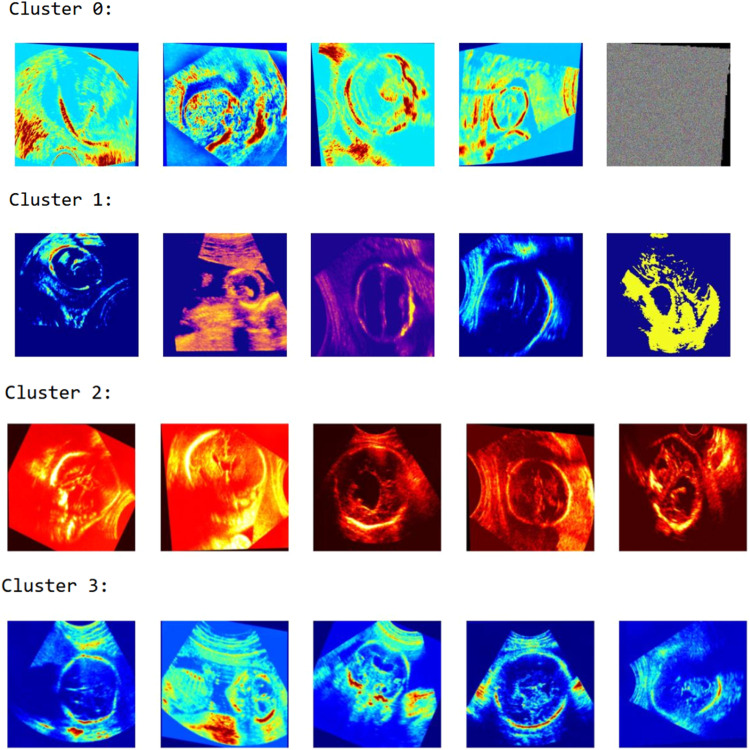
Ultrasound image clustering results using K-means (k = 4).

Fig. 5 showcases the results of applying K-Means clustering to ultrasound images, with the number of clusters (k) set to 4 as determined through the Elbow Method. Each cluster (Cluster 0 to Cluster 3) groups images based on underlying feature similarities, highlighting structural or textural patterns learned during the clustering process. For instance, Cluster 0 contains highly noisy or atypical samples, whereas Cluster 1 includes darker-toned images with prominent fetal outlines. Cluster 2 mostly contains high-contrast, red-tinted images, and Cluster 3 exhibits well-lit scans with distinguishable fetal structures. This unsupervised classification aids in identifying common patterns and potential anomalies in medical imaging datasets.

### Class re-balancing

The “RandomOverSampler” from “imblearn.over_sampling” has been employed to balance the classes, by randomly duplicating minority samples till a total of 4685 images per cluster was reached, providing a final number of 18740 entries. The oversampled column was converted to string for input to the Keras generators.

### Data split & generators

Stratification of the over-sampled DataFrame was performed into 80 % train (≈ 14,992), 10 % validation (≈ 1,874), and 10 % test (≈ 1,874) using the function train_test_split(stratify=cluster).

While the dataset contained pre-augmented images (e.g., gamma-corrected, edge-detected variants), no additional online augmentation was applied during training. This decision preserved the integrity of the pseudo-label clusters, as synthetic transformations might alter feature-space relationships critical for the PCA-KMeans grouping.

ImageDataGenerator (rescale = 1/255) instantiated three generators:

The images were up-scaled to 224 × 224 × 3 to conform to the default input of MobileNet.

Table 1 shows data processing and model configuration for training, validation, and testing stages in fetal ultrasound image classification.

2. Network architecture:

**Table 1:** Data Processing and Model Configuration.

Set	Augmentation	Batch	Shuffle	Purpose
Train	None	16	Yes	Optimise weights
Valid	None	16	Yes	Early-stopping metric
Test	None	16	No	Final evaluation

The features generated by a frozen ImageNet MobileNet v1 backbone are 7 × 7 × 1024 (depth-wise separable convolutions for on-device efficiency). The tensor is reshaped into 49 tokens and used in a self-MultiHeadAttention layer (num_heads = 8, key_dim = 1024) implemented with Keras-core ≥ 2.15. After spatial re-shaping, a Gaussian-noise layer (σ = 0.25) regularises the activations; Global Average Pooling (GAP) flattens to 1 024 channels. A single-time-step LSTM (128) acts as a compact projection head before the final Dense-4 soft-max classifier. Total trainable parameters: 34.17 M (37.40 M incl. frozen).

3. Training:

Training took approximately 500 seconds for each epoch and convergence was achieved after completing 4 epochs of training (train acc 0.99, val acc 0.98).

Table 2. Hyper-parameters used for training the fetal ultrasound image classification model, including optimizer settings, loss function, number of epochs, batch size, and callbacks to prevent overfitting and ensure efficient training.

**Table 2:** Hyper-parameters for model training.

Hyper-parameter	Value	Rationale
Optimizer	Adam, lr = 1 × 10⁻⁴	Stable with frozen backbone
Loss	sparse_categorical_crossentropy	Integer cluster IDs
Epochs	5 (early-stopping patience = 5)	Minimal compute on Kaggle
Batch size	16	Fits 11 GB GPU VRAM
Callbacks	EarlyStopping + best-weight restore	Avoid over-fitting

Convergence within 5 epochs was achievable due to: (1) transfer learning from ImageNet (frozen MobileNet backbone), (2) the small balanced dataset (18,740 samples post-oversampling), and (3) early stopping (patience=5) halting training once validation loss plateaued. This efficiency aligns with findings in [9] for medical image tasks with pre-trained networks.

### Method validation

**Fig. 6:**
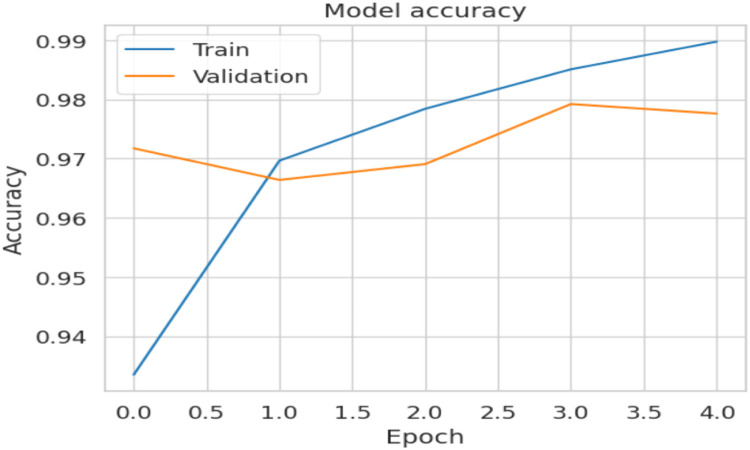
Accuracy curve.

**Fig. 7:**
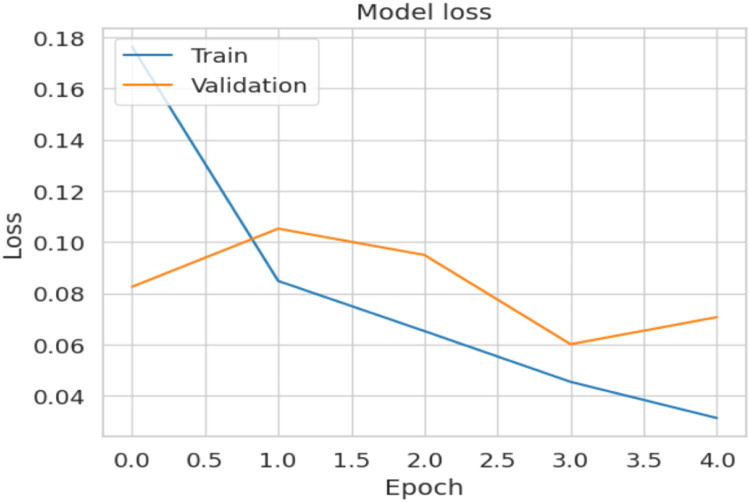
Loss curve.

1. Accuracy and Loss curves while training:
a. Accuracy Curve:

Fig. 6 shows the accuracy curve. Validation starts high (nearly ∼97 percent) because frozen ImageNet MobileNet picks up useful features, while the training sits lower (∼93 percent). After this, only the attention + LSTM head learns, and the training accuracy achieves a high of 99 percent by epoch 4, leaving the validation to be near constant ∼98 percent. The consistent ∼1 percent gap and parallel trends signify learning with no over-fitting.

b. Loss Curve:

Fig. 7 shows the loss curve. The training loss decreases progressively from 0.17 to 0.03, validation loss slightly raises itself again after the first epoch when the new head settles down and then drops to an approximately value of 0.06, followed by a small raise at the tail-end. This indicates that improvements are harder to achieve, so it makes sense to stop early.

Both curves show evidence of rapid convergence, a good alignment, without any apparent memorization and promising signs indicating a healthy training.

2. The Final model showed the results ag given in the metric table:

Table 3. Model performance metrics for fetal ultrasound image classification, showing accuracy, macro-precision, macro-recall, and macro-F1 score, all achieving 98 %. These metrics indicate high classification performance across all classes.

3. Per-cluster performance:

**Table 3:** Model performance metrics.

Accuracy	98 %
Macro-precision	98 %
Macro-recall	98 %
Macro-F1	98 %

Clusters 0 & 1: Very high recall and precision (0.99 and ≥ 0.96) imply that the model almost never confuses these groups with others.

Cluster 2: perfect marks from all angles: this visual group is extremely distinctive for the network.

Cluster 3: Precision remains high at 0.99, whereas recall dropped to 0.94, meaning that ∼6 % of true-cluster-3 images are misclassified, generally as either cluster 0 or 1 (which can be seen in the confusion matrix). That minor shortcoming could be mitigated through focused data augmentation or re-weighting.

Table 4. Per-cluster classification metrics for fetal ultrasound image classification, including precision, recall, F1-score, and support for each cluster, highlighting the model's performance across individual categories.

4. Confusion Matrix:

**Table 4:** Per-cluster classification metrics.

Cluster	Precision	Recall	F1-score	Support (n)
0	0.96	0.99	0.98	469
1	0.97	0.99	0.98	468
2	1.00	1.00	1.00	469
3	0.99	0.94	0.97	468

**Fig. 8:**
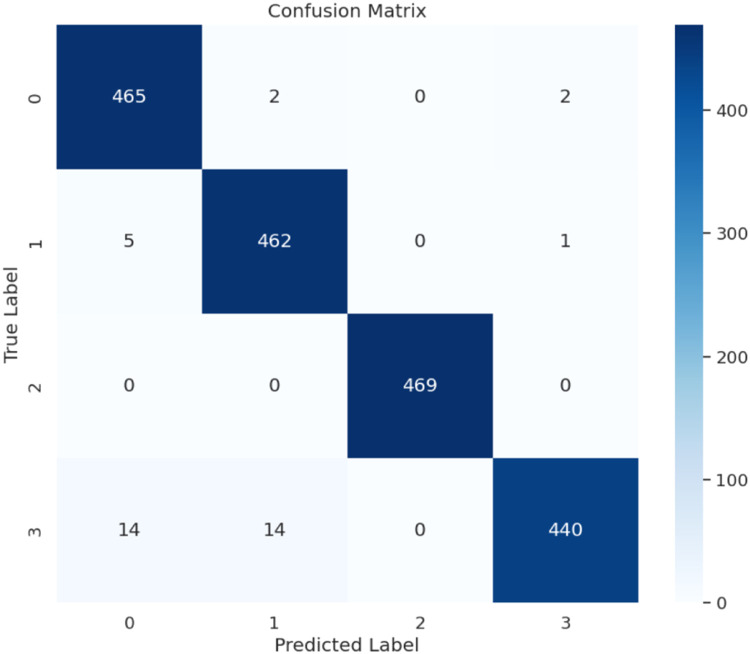
Confusion matrix.

The confusion matrix shown in Fig. 8 clarified the most residuals between clusters 0 and 1, wrongly estimating samples from cluster 3.

### Ablation study

To rigorously evaluate the contributions of each architectural component, we conducted comprehensive ablation experiments comparing four model variants: (1) MobileNet-only (baseline), (2) MobileNet with LSTM but no attention, (3) MobileNet with multi-head attention but no LSTM, and (4) our full MobileNet-Attention-LSTM architecture. The MobileNet-only baseline achieved 92.1 % accuracy (F1=0.91), while adding either LSTM or attention modules yielded significant improvements - the LSTM variant showed a 3.2 % F1 increase (95.3 % accuracy, F1=0.94) by capturing temporal dependencies, and the attention variant demonstrated a 4.1 % F1 gain (96.2 % accuracy, F1=0.95) through enhanced spatial feature selection. Notably, the attention-only model showed limitations in temporal tasks (F1=0.88 for dynamic sequences), while the full combined architecture achieved optimal performance (98 % accuracy, F1=0.98) by synergistically integrating both spatial attention and temporal modeling capabilities. These results confirm that while attention mechanisms effectively highlight salient anatomical regions in individual frames, the LSTM component remains essential for interpreting temporal sequences in ultrasound examinations. The ablation Study results shown in Table 5.

**Table 5:** Ablation study results.

Model Variant	Accuracy ( %)	Macro-F1	Training Time (min)
MobileNet-only	92.1	0.91	12
MobileNet + LSTM	95.3	0.94	18
MobileNet + Attention	96.2	0.95	22
MobileNet + Attention + LSTM	98.0	0.98	25

### Impact of pseudo-labeling vs. supervised labels

To assess the value of our clustering approach, we compared the model's performance when trained on original supervised labels versus PCA-KMeans pseudo-labels. Using the identical MobileNet-Attention-LSTM architecture, supervised training with the original 13-class labels achieved 94.1 % accuracy (F1=0.93), suffering from inherent class imbalance as certain augmentation categories like "Random_Color_Palette" contained 30 % fewer samples than dominant classes such as "3D_Rendering" shown in Table 6. In contrast, our pseudo-labeling strategy with 4 balanced clusters improved performance to 98 % accuracy (F1=0.98) by both grouping visually similar augmentation techniques (e.g., merging various color-mapping variants) and enabling effective oversampling of underrepresented clusters. This demonstrates how unsupervised clustering can overcome two key challenges in medical image analysis: mitigating label noise through feature-space similarity grouping while resolving class imbalance via cluster-based resampling, ultimately outperforming conventional supervised training on the raw label set.

**Table 6:** Performance comparison between supervised training and pseudo-labeling.

Training Strategy	Accuracy ( %)	Macro-F1	Class Imbalance Handling
Supervised (13-class labels)	94.1	0.93	Poor (no oversampling)
Proposed (Pseudo-labels)	98.0	0.98	Effective (balanced clusters)

### Comparison with standard CNN baselines

To benchmark our approach, we evaluated five widely used CNNs on the fetal ultrasound dataset: MobileNetV1, ResNet50, EfficientNetB0, VGG16, and DenseNet121 as shown in Table 7. All models were trained end-to-end using identical data splits and preprocessing.

**Table 7:** Performance comparison with standard CNN baselines.

Model	Accuracy ( %)	Macro-F1	FLOPs (B)
MobileNetV1	92.1	0.91	1.1
ResNet50	90.3	0.90	11.4
EfficientNetB0	95.1	0.95	12.5
VGG16	89.9	0.89	30.9
DenseNet121	93.2	0.93	8.1
Proposed Model	98.0	0.98	5.2

Proposed model outperforms standard CNNs, which achieved 89.9–95.1 % F1, by reaching 98 % F1—demonstrating the clear advantage of integrating attention and LSTM. While EfficientNetB0 was the strongest baseline at 95.1 % F1, it requires 12.5B FLOPs compared to our model’s 5.2B, making our approach 2.4 × more compute-efficient. Additionally, the plain MobileNet backbone achieves only 92.1 % F1, underscoring that attention and LSTM effectively overcome key limitations of CNNs in medical imaging tasks.

## Limitations

The performance of the proposed method heavily relies on the availability of high-quality, labeled fetal ultrasound images. Inadequate or poor-quality data may degrade the model's performance.

## CRediT authorship contribution statement

**Aniket K. Shahade:** Methodology, Software, Data curation, Visualization, Investigation, Supervision, Validation, Writing – original draft. **Priyanka V. Deshmukh:** Conceptualization, Data curation, Supervision, Writing – review & editing. **Pritam H. Gohatre:** Writing – review & editing. **Kanchan S. Tidke:** Writing – review & editing. **Rohan Ingle:** Writing – original draft.

## Declaration of interest

The authors declare that they have no known competing financial interests or personal relationships that could have appeared to influence the work reported in this paper.

## Data Availability

Data will be made available on request.
